# Supplementary figures and images for: The molecular basis for DNA-binding by competence T4P is distinct in a representative Gram-positive and Gram-negative species
Source: PLoS Pathog. 2025 Apr 21;21(4):e1013128. doi: 10.1371/journal.ppat.1013128 (PMC12040237; doi:10.1371/journal.ppat.1013128)

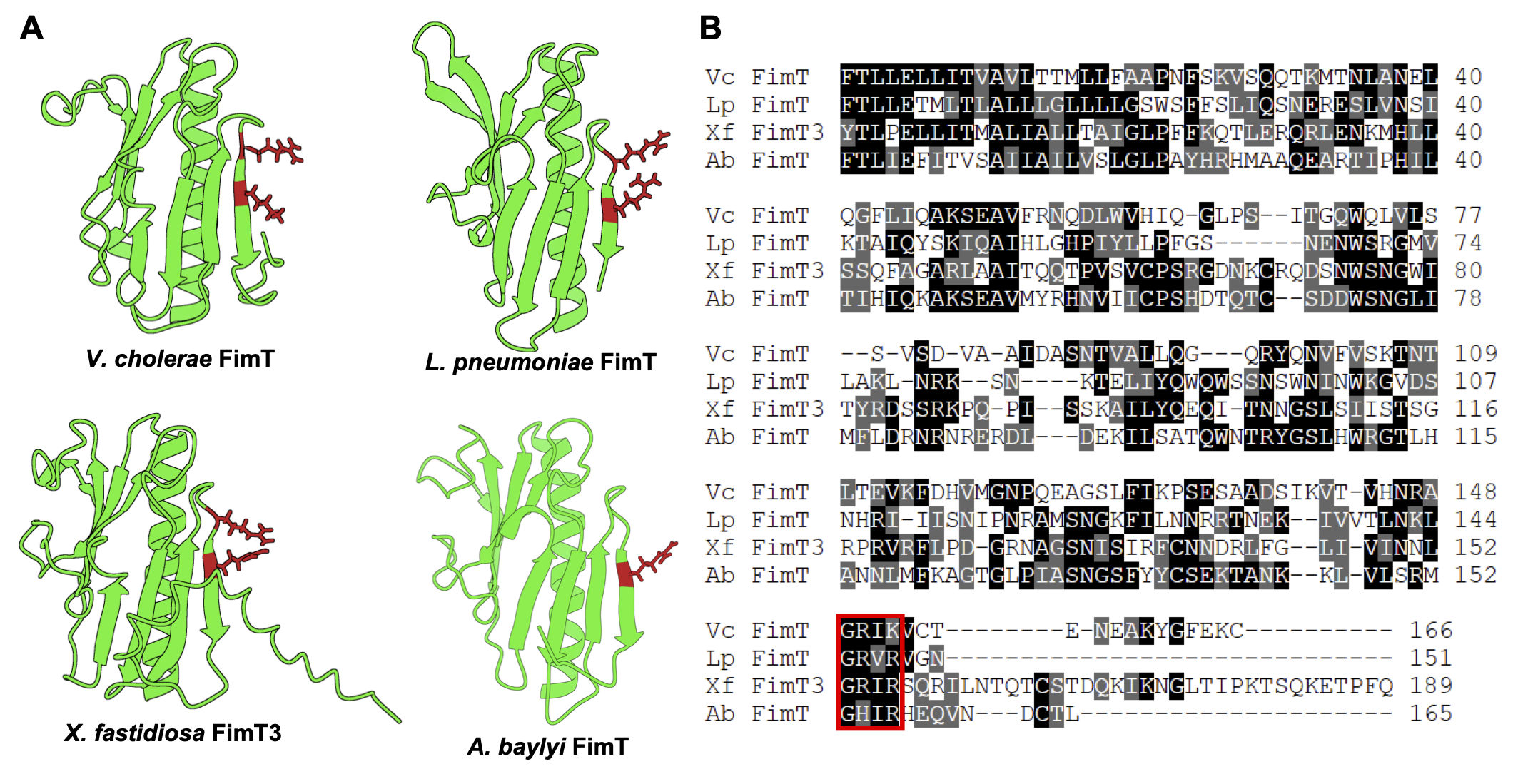

Supplement: S1 Fig — The R/K residues from the conserved G-[R/K]-X-[R/K] motif that are required for DNA binding are highlighted in red. (B) Multiple sequence alignment of FimT homologs, (Vc, V. cholerae; Lp, L. pneumophilla; Xf, X. fastidiosa; Ab, A. baylyi). The G-[R/K]-X-[R/K] motif in each homolog is boxed in red. Residues that are identical are shown in black, while residues that are similar are shown in gray. (TIFF) [file ppat.1013128.s001.tiff]

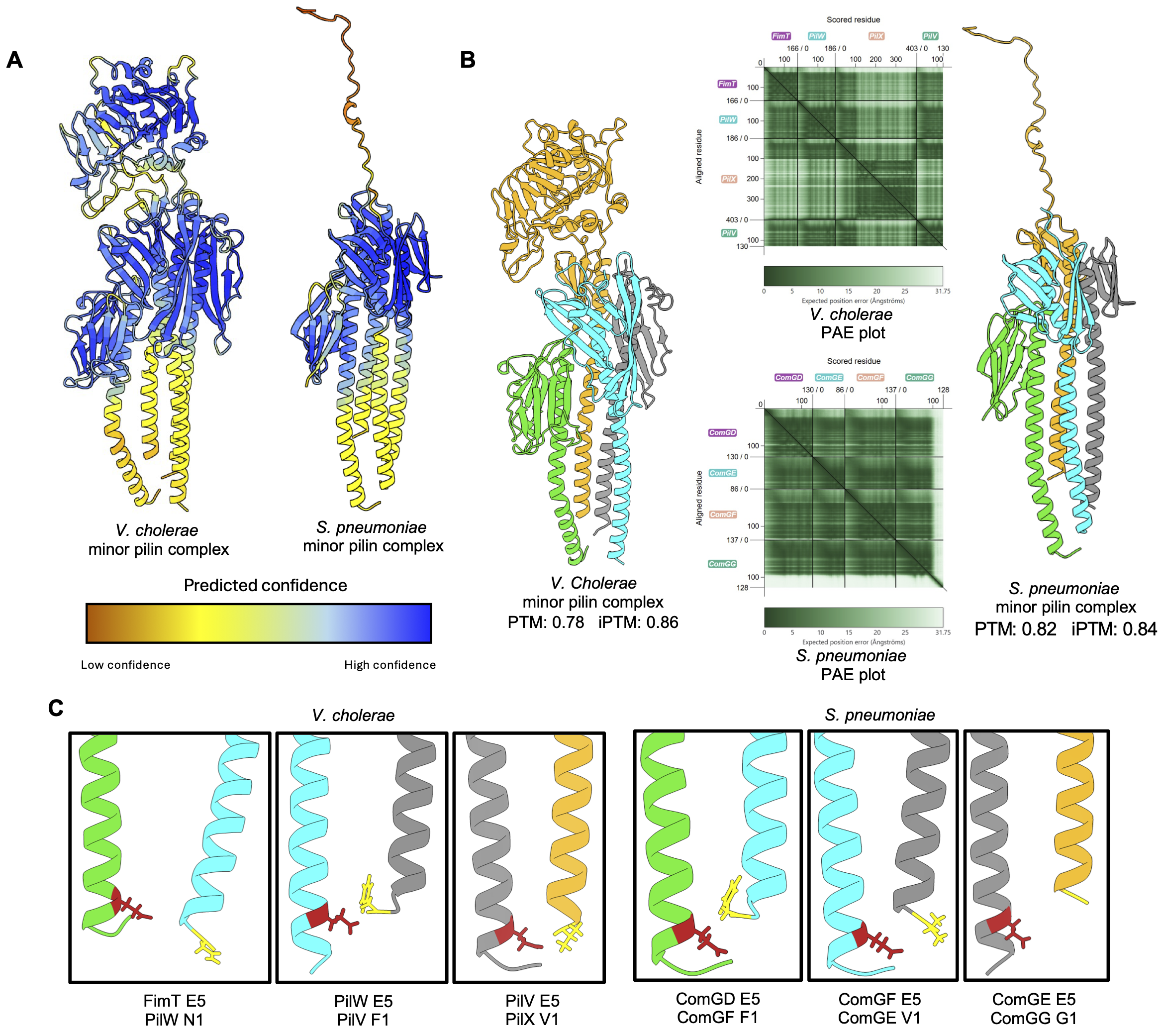

Supplement: S2 Fig — (A) Structural predictions of competence minor pilin tip complexes from V. cholerae and S. pneumoniae colored by predicted confidence (pLDDT). (B) Competence minor pilin tip complexes as in (A) but colored by minor pilin: FimT (VC0858/ ComGD) in green, PilW (VC0859/ ComGF) in cyan, PilV (VC0861/ ComGE) in gray, and PilX (VC0860/ ComGG) in yellow. Also included are the pTM and ipTM scores as well as the PAE plots for each model. (C) Predicted E5 interactions between competence minor pilins within the complex. The E5 residue is colored red while the N-terminal residue of the neighboring pilin is colored yellow. (TIFF) [file ppat.1013128.s002.tiff]

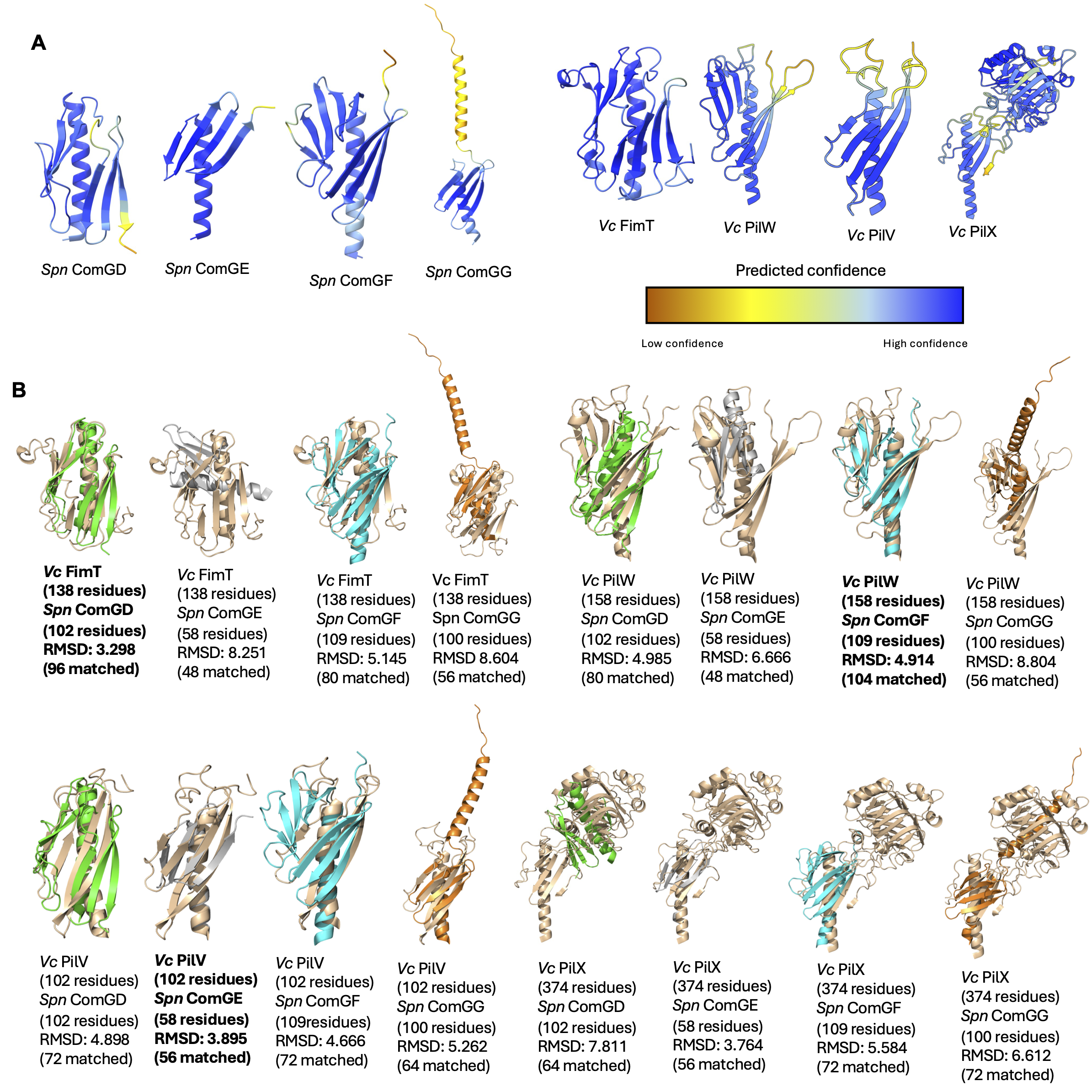

Supplement: S3 Fig — (A) Individual competence minor pilins from S. pneumoniae (Spn) and V. cholerae (Vc) colored by predicted confidence score. (B) Alignment of the headgroups (i.e., lacking residues 1–28) of the indicated minor pilins. V. cholerae minor pilins are shown in tan, while S. pneumoniae minor pilins are colored (ComGD, green; ComGE, gray; ComGF, cyan; ComGG, orange). Captions for each pairing denote the proteins aligned, the number of residues in each minor pilin, the calculated RMSD value, and the number of residues matched during the RMSD calculation. Pilin pairings with the lowest RMSD were deemed structural homologs (denoted in bold) with the exception of the PilX/ ComGG pairing, which were deemed homologs based on the lack of an E5 (see text and S2 Fig for details). (TIFF) [file ppat.1013128.s003.tiff]

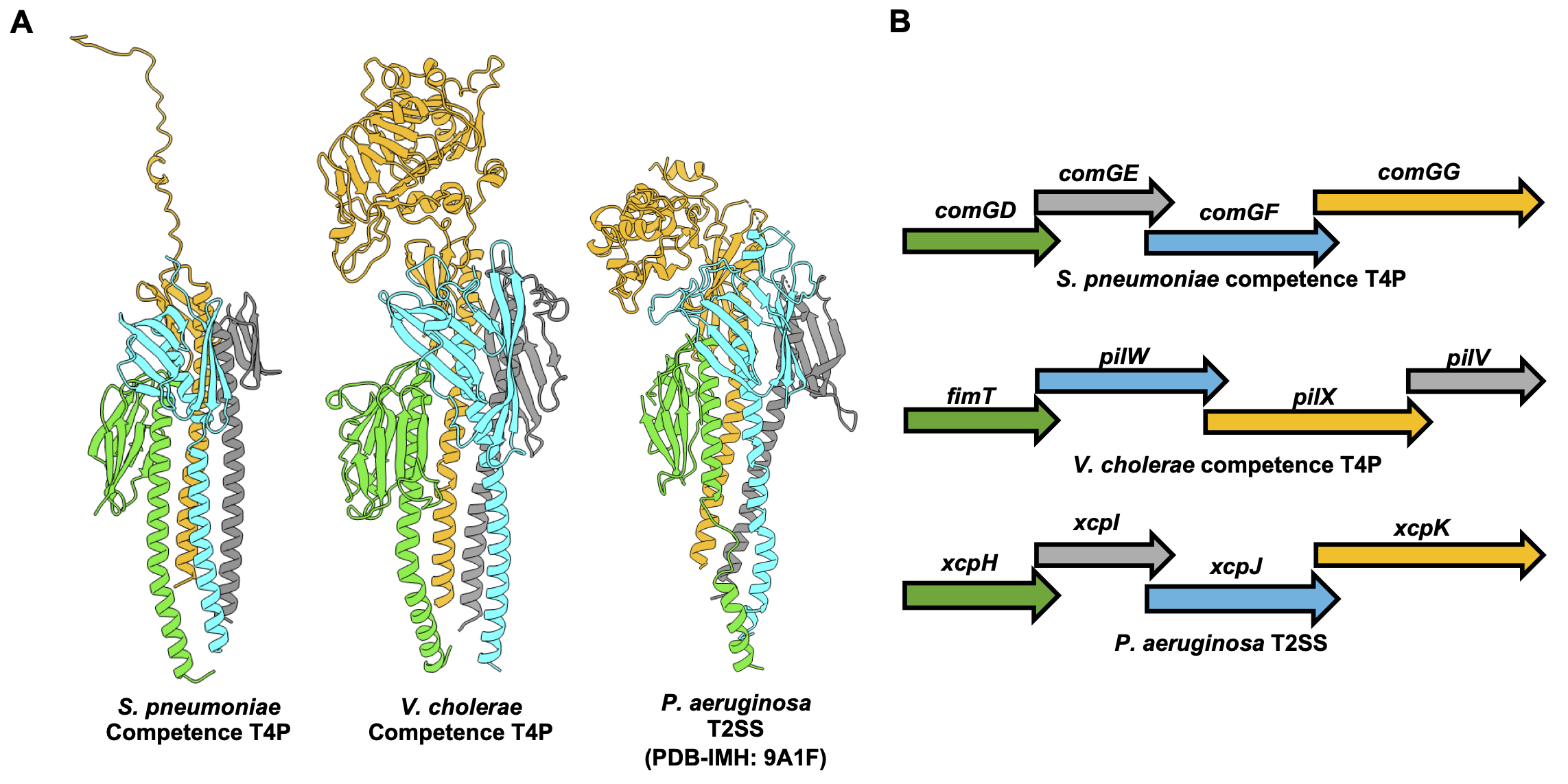

Supplement: S4 Fig — (A) AF-m models of the indicated minor pilin tip complexes and the integrative structural model of the P. aeruginosa T2SS minor pilin complex. (B) Schematic of the minor pilin operons for the indicated systems. Proteins in A and gene designations in B are color matched for ease of comparison. (TIFF) [file ppat.1013128.s004.tiff]

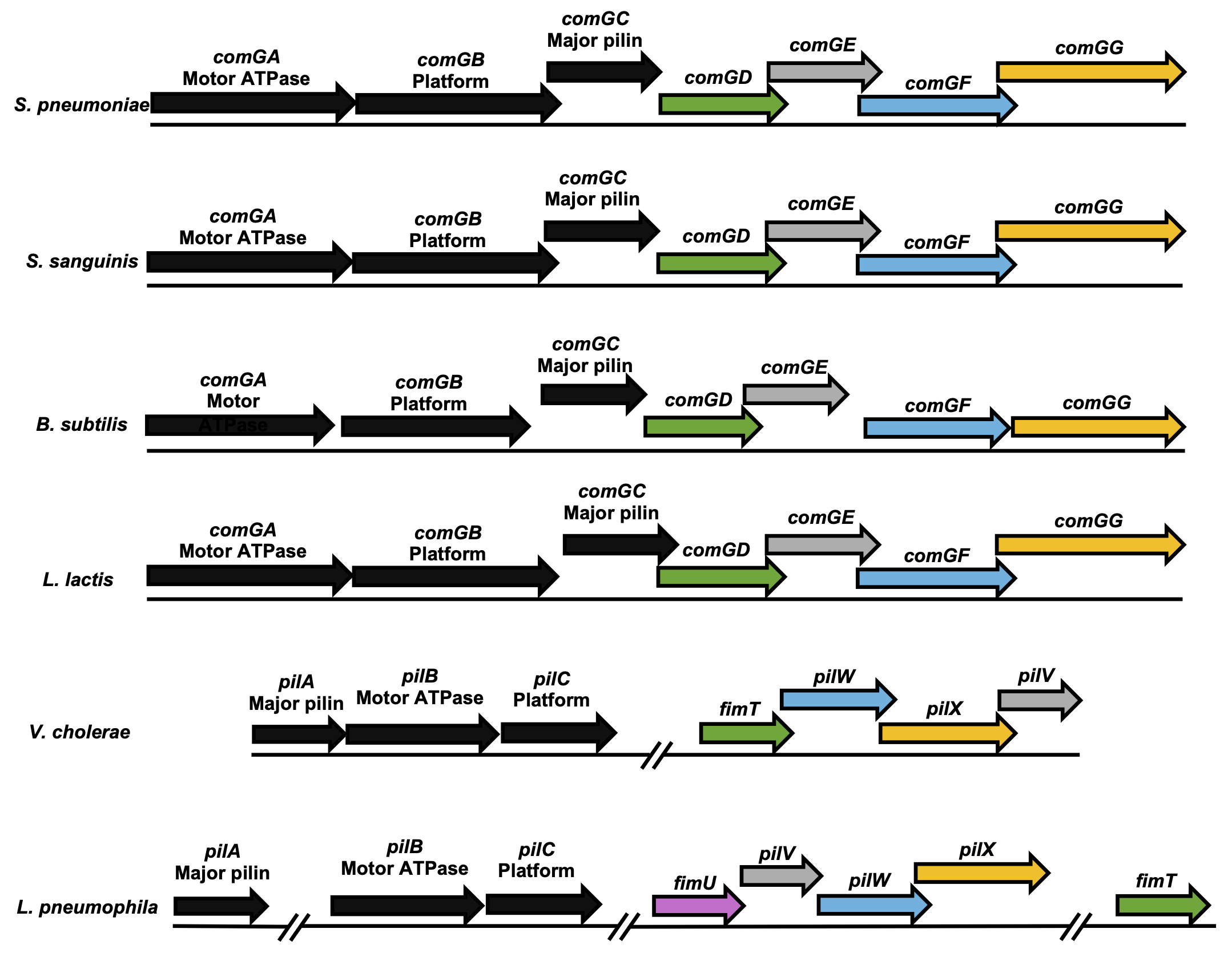

Supplement: S5 Fig — Gene arrangements of the minor pilins, major pilin, motor ATPase, and platform proteins in select monoderms and diderms. (TIFF) [file ppat.1013128.s005.tiff]

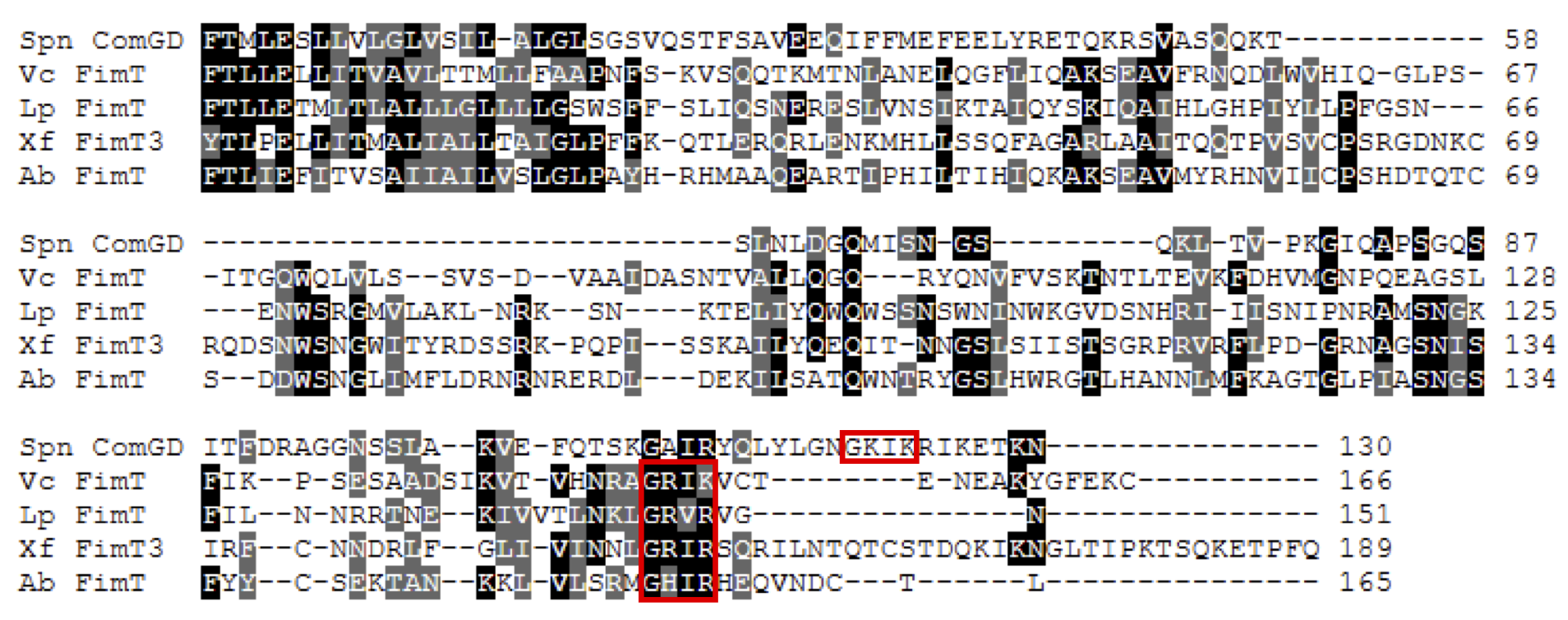

Supplement: S6 Fig — MSA of FimT homologs, (Spn, S. pneumoniae; Vc, V. cholerae; Lp, L. pneumophilla; Xf, X. fastidiosa; Ab, A. baylyi). The G-[R/K]-X-[R/K] motif in each homolog is boxed in red. Residues that are identical are shown in black, while residues that are similar are shown in gray. (TIFF) [file ppat.1013128.s006.tiff]

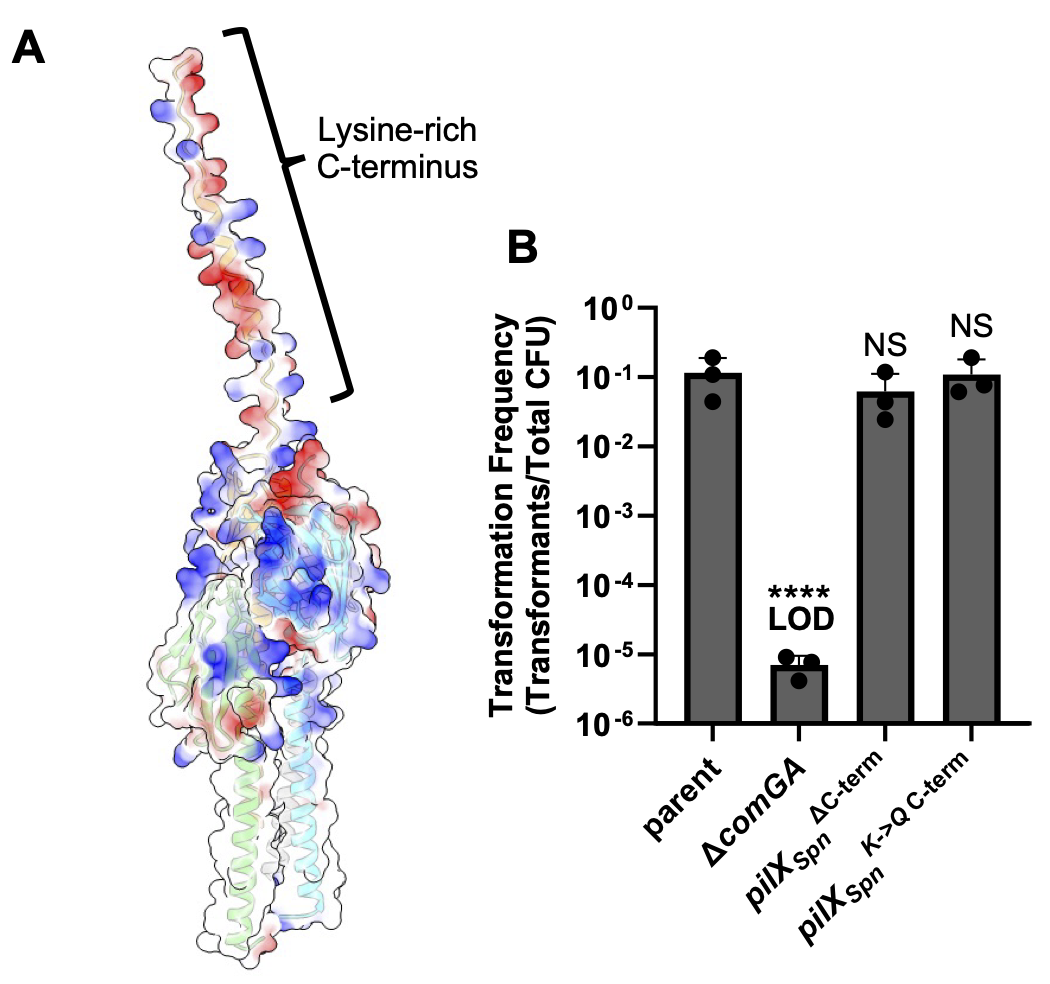

Supplement: S7 Fig — (A) Electrostatic surface map of the S. pneumoniae minor pilin tip complex highlighting the lysine-rich C-terminus. (B) NT assay of the indicated S. pneumoniae strains. In pilXSpnΔC-term, residues 101–137 were deleted. In pilXSpnK->Q C-term, all lysine residues within the C-terminus (residues 101–137) were mutated to glutamine (i.e., VKIKEEKRDKKEEVATDSSEKVEKKKSEEKPEKKENS was mutated to VQIQEEQRDQQEEVATDSSEQVEQQQSEEQPEQQENS). Data in B is from at least 3 independent biological replicates and shown as the mean ± SD. Statistical comparisons were made by one-way ANOVA with Turkey’s multiple comparison test of the log-transformed data. NS, no significance; **** = p < 0.0001. LOD, limit of detection. Statistical identifiers directly above bars represent comparisons to the parent. (TIFF) [file ppat.1013128.s007.tiff]

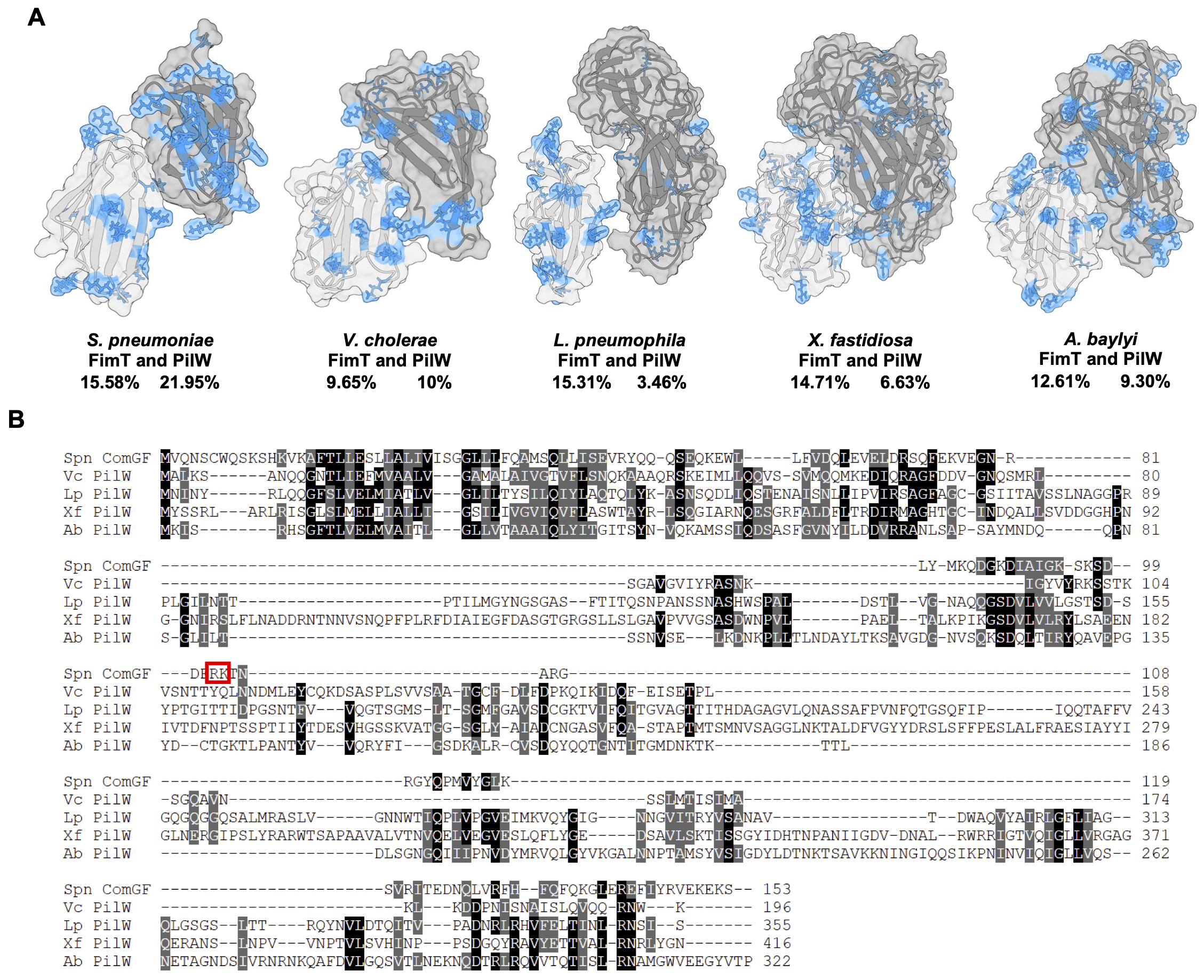

Supplement: S8 Fig — (A) Surface maps of FimT (light grey) and PilW (dark grey) homologs with arginine and lysine residues colored blue. The percent of surface residues that are arginines/lysines is indicated for each homolog. (B) MSAs of PilW homologs (Spn, S. pneumoniae; Vc, V. cholerae; Lp, L. pneumophilla; Xf, X. fastidiosa; Ab, A. baylyi). The residues shown to be important for DNA binding in S. pneumoniae are boxed in red. (TIFF) [file ppat.1013128.s008.tiff]

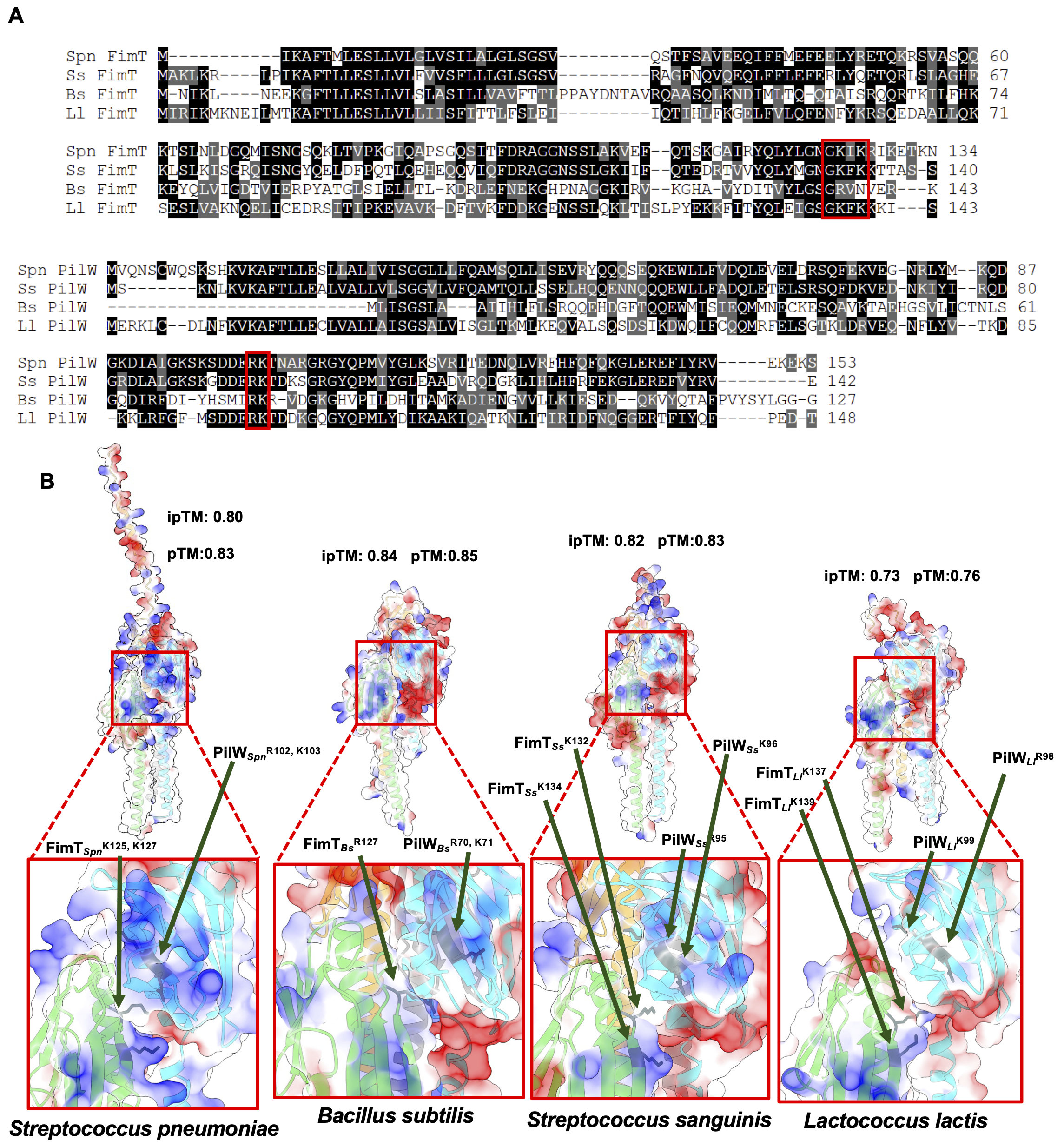

Supplement: S9 Fig — (A) MSAs of FimT (i.e., ComGD) and PilW (i.e., ComGF) homologs from four naturally competent monoderms. Spn, S. pneumoniae; Ss, S. sanguinis; Bs, B. subtilis; Ll, L. lactis. Red boxes indicate conserved residues shown to be important for NT and DNA binding in S. pneumoniae. Residues that are identical are shown in black, while residues that are similar are shown in gray. (B) Electrostatic surface maps of AlphaFold3 models of the indicated minor pilin tip complexes highlight the conserved positively-charged patch spanning FimT and PilW. Insets further highlight the positional conservation of the R/K residues shown to be critical for DNA-binding in FimTSpn and PilWSpn. (TIFF) [file ppat.1013128.s009.tiff]
