# Supplementary material for: The molecular basis for DNA-binding by competence T4P is distinct in a representative Gram-positive and Gram-negative species
Source: PLoS Pathog. 2025 Apr 21;21(4):e1013128. doi: 10.1371/journal.ppat.1013128 (PMC12040237; doi:10.1371/journal.ppat.1013128)
Supplement: S1 Table — (PDF) [file ppat.1013128.s010.pdf]

Table S1 – Strains used in this study

Strain #	Genotype	Figures	Use in manuscript
TND0905/ SAD2468	P_{tac}-*tfoX*, Δ*luxO*, *lacZ::lacI*^{q}, *pilA*^{S67C}, Δ*VC1807::Zeo*^{R}	Fig. 1A	Parent for all *V. cholerae* strains in this study and the parent for *V. cholerae* natural transformation assays
NDC073/ SAD3694	Δ*fimT*, P_{tac}-*tfoX*, Δ*luxO*, Δ*lacZ::Spec*^{R}, *pilA*^{S67C}, Δ*VC1807::Kan*^{R}	Fig. 1A	Δ*fimT* for *V.cholerae* natural transformation assays
NDC0429/ SAD3695	*fimT*^{R154Q}, P_{tac}-*tfoX*, Δ*luxO*, *lacZ::lacI*^{q}, *pilA*^{S67C}, Δ*VC1807::Spec*^{R}	Fig. 1A	*fimT*^{R154Q} for *V.cholerae* natural transformation assays
NDC0413/ SAD3696	*fimT*^{K156Q}, P_{tac}-*tfoX*, Δ*luxO*, *lacZ::lacI*^{q}, *pilA*^{S67C}, Δ*VC1807::Spec*^{R}	Fig. 1A	*fimT*^{K156Q} for *V.cholerae* natural transformation assays
TND1035/ SAD2469	Δ*pilT::Tm*^{R}, P_{tac}-*tfoX*, Δ*luxO*, *lacZ::lacI*^{q}, *pilA*^{S67C}, Δ*VC1807::Zeo*^{R}	Fig. 1B, C	Parent for *V. cholerae* DNA-binding assay and surface piliation microscopy
NDC074/ SAD3697	Δ*fimT*, Δ*pilT::Tm*^{R} P_{tac}-*tfoX*, Δ*luxO*, Δ*lacZ::Spec*^{R}, *pilA*^{S67C}, Δ*VC1807::Kan*^{R}	Fig. 1B, C	Δ*fimT* for *V.cholerae* DNA-binding assay and surface piliation microscopy
NDC0453/ SAD3698	*fimT*^{R154Q}, Δ*pilT::Tm*^{R} P_{tac}-*tfoX*, Δ*luxO*, Δ*lacZ::Spec*^{R}, *pilA*^{S67C}, Δ*VC1807::Kan*^{R}	Fig. 1B, C	*fimT*^{R154Q} for *V.cholerae* DNA-binding assay and surface piliation microscopy
NDC0441/ SAD3699	*fimT*^{K156Q}, Δ*pilT::Tm*^{R} P_{tac}-*tfoX*, Δ*luxO*, Δ*lacZ::Spec*^{R}, *pilA*^{S67C}, Δ*VC1807::Kan*^{R}	Fig. 1B, C	*fimT*^{K156Q} for *V.cholerae* DNA-binding assay and surface piliation microscopy
NDC0287/ SAD3474	P_{cilA}-*comGC-FLAG* downstream of treR *Kan*^{R}, Δ*comC*	Fig. 2A, 3B-C, 4, S3	Strain RL001. Parent of all *S. pneumoniae* strains in this study and the parent for all *Spn* transformation assays, western blot analysis, and DNA-binding assay
NDC0326/ SAD3700	Δ*comGA::Erm*^{R}, P_{cilA}-*comGC-FLAG* downstream of treR *Kan*^{R}, Δ*comC*	Fig. 2A, 3B-C, 4, S3, S4	Δ*comGA* for all *Spn* transformation assays, western blot analysis, and DNA-binding assays
NDC0449/ SAD3701	Δ*fimT*$_{Spn}$, P_{cilA}-*comGC-FLAG* downstream of treR *Kan*^{R}, Δ*comC*	Fig. 2A, 3B-C	Δ*fimT*$_{Spn}$ for all *Spn* transformation assays, and western blot analysis
NDC0456/ SAD3702	*fimT*$_{Spn}^{K125Q}$, P_{cilA}-*comGC-FLAG* downstream of treR *Kan*^{R}, Δ*comC*	Fig. 2A	*fimT*$_{Spn}^{K125Q}$ for *Spn* transformation assays
NDC0457/ SAD3703	*fimT*$_{Spn}^{K127Q}$, P_{cilA}-*comGC-FLAG* downstream of treR *Kan*^{R}, Δ*comC*	Fig. 2A	*fimT*$_{Spn}^{K127Q}$ for *Spn* transformation assays
NDC0431/ SAD3704	*fimT*$_{Spn}^{K125Q,\ K127Q}$, P_{cilA}-*comGC-FLAG* downstream of treR *Kan*^{R}, Δ*comC*	Fig. 2A, 3B-C	*fimT*$_{Spn}^{K125Q,\ K127Q}$ for *Spn* transformation assays and western blots
NDC0432/ SAD3705	*pilW*$_{Spn}^{R102Q}$, P_{cilA}-*comGC-FLAG* downstream of treR *Kan*^{R}, Δ*comC*	Fig. 3B-C	*pilW*$_{Spn}^{R102Q}$ for *Spn* transformation assays and western blots
NDC0443/ SAD3706	*fimT*$_{Spn}^{K125Q,\ K127Q}$, *pilW*$_{Spn}^{R102Q}$, P_{cilA}-*comGC-FLAG* downstream of treR *Kan*^{R}, Δ*comC*	Fig. 3B	*fimT*$_{Spn}^{K125Q,\ K127Q}$, *pilW*$_{Spn}^{R102Q}$ for *Spn* transformation assays
NDC0466/ SAD3708	*fimT*$_{Spn}^{R116Q}$, P_{cilA}-*comGC-FLAG* downstream of treR *Kan*^{R}, Δ*comC*	Fig. 3B	*fimT*$_{Spn}^{R116Q}$ for *Spn* transformation assays
NDC0483/ SAD3708	Δ*pilW*$_{Spn}$, P_{cilA}-*comGC-FLAG* downstream of treR *Kan*^{R}, Δ*comC*	Fig. 3B-C	Δ*pilW*$_{Spn}$ for *Spn* transformation assays, and western blots
NDC0484/ SAD3709	*pilW*$_{Spn}^{R102Q,\ K103Q}$, P_{cilA}-*comGC-FLAG* downstream of treR *Kan*^{R}, Δ*comC*	Fig. 3B-C	*pilW*$_{Spn}^{R102Q,\ K103Q}$ for *Spn* transformation assays and western blots
NDC0486/ SAD3710	*pilW*$_{Spn}^{K103Q}$, P_{cilA}-*comGC-FLAG* downstream of treR *Kan*^{R}, Δ*comC*	Fig. 3B-C	*pilW*$_{Spn}^{K103Q}$ for *Spn* transformation assays and western blots
NDC0487/ SAD3711	*fimT*$_{Spn}^{K105Q}$, P_{cilA}-*comGC-FLAG* downstream of treR *Kan*^{R}, Δ*comC*	Fig. 3B	*fimT*$_{Spn}^{K105Q}$ for *Spn* transformation assays
NDC0488/ SAD3712	*pilW*$_{Spn}^{K107Q}$, P_{cilA}-*comGC-FLAG* downstream of treR *Kan*^{R}, Δ*comC*	Fig. 3B	*pilW*$_{Spn}^{K107Q}$ for *Spn* transformation assays

NDC0489/ SAD3713	$pilW_{Spn}^{K109Q}$, P_{cilA}-*comGC-FLAG* downstream of treR Kan^{R}, Δ*comC*	Fig. 3B	$pilW_{Spn}^{K109Q}$ for *Spn* transformation assays
NDC0490/ SAD3714	$fimT_{Spn}^{K125Q,\ K127Q}$, $pilW_{Spn}^{K103Q}$, P_{cilA}-*comGC-FLAG* downstream of treR Kan^{R}, Δ*comC*	Fig. 3B	$fimT_{Spn}^{K125Q,\ K127Q}$, $pilW_{Spn}^{K103Q}$ for *Spn* transformation assays
NDC0491/ SAD3715	$fimT_{Spn}^{K125Q,\ K127Q}$, $pilW_{Spn}^{R102Q,\ K103Q}$, P_{cilA}-*comGC-FLAG* downstream of treR Kan^{R}, Δ*comC*	Fig. 3B-C, Fig 4, S3	$fimT_{Spn}^{K125Q,\ K127Q}$, $pilW_{Spn}^{R102Q,\ K103Q}$ for *Spn* transformation assays, western blots, and DNA-binding assays
NDC0430/ SAD3716	$comGC^{S66C}$, P_{cilA}-*comGC-FLAG* downstream of treR Kan^{R}, Δ*comC*	Fig. S4	Parent for *Spn comGG* transformation assays
NDC0494/ SAD3717	$comGG^{\Delta 101\text{-}137}$, $comGC^{S66C}$, P_{cilA}-*comGC-FLAG* downstream of treR Kan^{R}, Δ*comC*	Fig. S4	$comGG^{\Delta C\text{-}term}$ for *Spn* transformation assays
NDC0495/ SAD3718	$comGG^{K\text{->}Q}$, $comGC^{S66C}$, P_{cilA}-*comGC-FLAG* downstream of treR Kan^{R}, Δ*comC*	Fig. S4	*comGG* C-term K->Q for *Spn* transformation assays

***Double identifiers under "Strain #" refers to the same strain that has been stocked in two independent strain collections.**
