# Supplementary material for: The molecular basis for DNA-binding by competence T4P is distinct in a representative Gram-positive and Gram-negative species
Source: PLoS Pathog. 2025 Apr 21;21(4):e1013128. doi: 10.1371/journal.ppat.1013128 (PMC12040237; doi:10.1371/journal.ppat.1013128)
Supplement: S2 Table — (PDF) [file ppat.1013128.s011.pdf]

Table S2 – Primers used in this study

Primer	Sequence (5'→3')	Description
NCP0109	CGACTCCACAATTGTTGAAAGC	comGD F1
NCP0110	GTTCAATCAAACCTTTCGCCAATAC	comGD R2
NCP0152	GAAGAACTCTATCGGGAAACC	comGD detectF
NCP0143	CTTAGCTACCCTCAAGACTTCTTC	comGD detectR
NCP0168	GAAATTCAACtTgAGCCAGGGACGAATTGCCCCCAGCTC	comGD K105Q R1
NCP0115	CCCTGGCTcAaGTTGAATTTCAGACCAGTAAAGGAGCGATTCG	comGD K105Q F2
NCP0116	CTGGTCTGAAATTCAACtcg	comGD K105Q detect
NCP0117	CCTAGATATAATTGATAttGAATCGCTCCTTTACTGGTCTGAAATTCAACCTTAGC	comGD R116Q R1
NCP0294	GACCAGTAAAGGAGCGATTCaaTATCAATTATATCTAGGAAATGG	comGD R116Q F2
NCP0119	ACCAGTAAAGGAGCGATTtaa	comGD R116Q detect
NCP0120	GCGTTTAATcTgTCCATTTCCTAGATATAATTGATAGCGAATCGCTCC	comGD K125Q R1
NCP0121	CTAGGAAATGGAcAgATTAAACGCATTAAGGAAACAAAAAATTAGGGCAGTG	comGD K125Q F2
NCP0169	GTTTCCTTAATGCGTTTAATccg	comGD K125Q detect
NCP0123	CCTTAATGCGcTgAATTTTTCCATTTCCTAGATATAATTGATAGCGAATCG	comGD K127Q R1
NCP0124	GGAAAAATTcAgCGCATTAAGGAAACAAAAAATTAGGGCAGTGATTTTAC	comGD K127Q F2
NCP0170	TTTGTTTCCTTAATGCGccg	comGD K127Q detect
NCP0295	CTTAATGCGTTgAATTTgTCCATTTCCTAGATATAATTGATAGCGAATCGCTCC	comGD K125Q, K127Q R1
NCP0296	CTAGGAAATGGAcAAATTcAACGCATTAAGGAAACAAAAAATTAGGGCAGTG	comGD K125Q, K127Q F2
NCP0297	CAATTATATCTAGGAAATGGgcAAATcc	comGD K125Q, K127Q detect
NCP0126	GGCTATGAAAAACAAACCAGTCAG	comGF F1
NCP0127	CACGGTTAAGAACACGACTGATATC	comGF R2
NCP0145	ATTGTCATCAGTGGGGGATTAC	comGF detectF
NCP0144	CTTTCTTGCTCAACCTTATCTTTGG	comGF detectR
NCP0131	GAGCATTCGTTTTctGGAAATCATCTGACTTTGACTTACCGATGGC	comGF R102Q R1
NCP0132	GATGATTTCCagAAAACGAATGCTCGTGGTCGAGGTTATCAGCCT	comGF R102Q F2
NCP0133	TAAGTCAAAGTCAGATGATTTCtag	comGF R102Q detect
NCP0368	GAGCATTCGTcTgACGGAAATCATCTGACTTTGACTTACCGATGGC	comGF K103Q R1
NCP0369	TGATTTCCGTcAgACGAATGCTCGTGGTCGAGGTTATCAGCCTATG	comGF K103Q F2
NCP0370	AGTCAGATGATTTCCGTcgg	comGF K103Q detect
NCP0134	TAACCTCGACCctGAGCATTCGTTTTACGGAAATCATCTGACTTTG	comGF R107Q R1
NCP0135	GAATGCTCagGGTCGAGGTTATCAGCCTATGGTTTATGGACTC	comGF R107Q F2

NCP0136	GGCTGATAACCTCGACtct	comGF R107Q detect
NCP0137	CTGATAACCctGACCACGAGCATTCGTTTTACGGAAATCATCTG	comGF R109Q R1
NCP0138	CTCGTGGTCagGGTTATCAGCCTATGGTTTATGGACTCAAATCTG	comGF R109Q F2
NCP0139	AACGAATGCTCGTGGTtag	comGF R109Q detect
NCP0371	GAGCATTCGTcTgctGGAAATCATCTGACTTTGACTTACCGATGGC	comGF R102Q, K103Q R1
NCP0372	GATGATTTCCagcAgACGAATGCTCGTGGTCGAGGTTATCAGCCTA	comGF R102Q, K103Q F2
NCP0373	AGTCAGATGATTTCCagcgg	comGF R102Q, K103Q detect
NCP0190	GCATGAGAGTTTGGTTATCCG	comGG F1
NCP0183	ACTTTCCCAACTCCTTGTATTG	comGG R2
NCP0221	CAACGCATTATAGTTGAATTGACTATGAAGGAAACAGAAACTCATATTG	comGG C-term deletion R1
NCP0222	TAGTCAATTCAACTATAATGCGTTG	comGG C-term deletion F2
NCP0223	AATCTAGGTCAGGTAAGCTATC	comGG C-term mod detectF
NCP0224	GATATAAACAAATGAATTGGAGCTTAAC	comGG C-term mod detectR
NCP0225	CACcTgTTCGCTTGAATCGGTCGCTACCTCTTCtTgcTgATCTCTcTgCTCTTCcTgGATcTgGACTGAAGG	comGG C-term K→Q R1
NCP0226	GTAGCGACCGATTCAAGCGAAcAgGTGGAGcAacAgcAgTCAGAAGAGcAaCCTGAAcAacAgGAGAATTCA	comGG C-term K→Q R1
NCP0233	TTCTCcTgtTgTTCAGGtcg	comGG C-term K→Q detectR1
NCP0234	ATTGACTATGAATTCTCcTgtcg	comGG C-term K→Q detectR2
NCP0235	TTCTGTTTCCTTCAGTCcgg	comGG C-term K→Q detectF1
NCP0236	AAGAGcAgAGAGATcAgcga	comGG C-term K→Q detectF2
NCP0179	gtcgacggatccccggaatCATTGACTTTACGATTTGCTCC	ΔcomGD R1
NCP0180	gaagcagctccagcctacaCGCTATCAATTATATCTAGGAAATGG	ΔcomGD F2
NCP0181	CAAGACTTCTTCCTTTTGCAAG	ΔcomGD detect
NCP0382	gtcgacggatccccggaatGACCTTATGGCTCTTTGATTG	ΔcomGF R1
NCP0383	agaagcagctccagcctacaGGCTTAGAAAGGGAGTTCATC	ΔcomGF F2
NCP0174	AGGAGGTTAACATTATGCCTAAC	ΔcomGA F1
NCP0175	gtcgacggatccccggaatCATACTTACCTCCTCACCTATAC	ΔcomGA R1
NCP0176	gaagcagctccagcctacaAAAGATGGACATATCACAAGTC	ΔcomGA F2
NCP0177	GATATAAACAAATGAATTGGAGCTTAAC	ΔcomGA R2
NCP0178	TTCTGAGAATGATTTCCCCTGAG	ΔcomGA detect
DOG0549	AACGACATTACTGACGTTATCCTAG	ΔfimT F1
DOG0550	gtcgacggatccccggaatATGCATTTCCCAAAATCCTTTGC	ΔfimT R1
DOG0551	gaagcagctccagcctacaGCTGGACGAATCAAAGTTTGC	ΔfimT F2
DOG0552	AATGATACCGCCCAGAGGAATC	ΔfimT R2
DOG0553	ATCACACCGACAGCACCA	ΔfimT detect
CE354	CTGCAGATTCAATCAAAGTGACTGTGCACAATCGGGCTGGACagATCAAAGTTTGCACC	FimT R154Q F2
CE355	AGTCACTTTGATTGAATCTGCAG	FimT R154Q R2
CE356	CGGTGCAAACTTTGAcct	FimT R154Q detect

NCP0316	GCAAACTTgGATTCGTCCAGCCCGATTGTGCACAGTCAC	FimT K156Q R1
NCP0317	CTGGACGAATCcAAGTTTGCACCGAAAATGAGGCAAAATATGG	FimT K156Q F2
NCP0318	CATTTTCGGTGCAAACTcg	FimT K156Q detect
DOG0400	ACTTCTGGCTGAAGGTCAATTTTC	ΔpilT F1
DOG0401	gtcgacggatccccggaatCATTTAAATTCCTTAATAAAGTCTGGC	ΔpilT R1
DOG0402	gaagcagctccagcctacaTAGGTAGGTAAAGACAGATGGAG	ΔpilT F2
DOG0403	TCACGTGTTCGGCCAAAATC	ΔpilT R2
DOG0404	TTCTGCTTGCCTTGCGTC	ΔpilT detect
